# Regulation of sensitivity of tumor cells to antitubulin drugs by Cdk1-TAZ signalling

**DOI:** 10.18632/oncotarget.4259

**Published:** 2015-06-08

**Authors:** Yulei Zhao, Xiaolong Yang

**Affiliations:** ^1^ Department of Pathology and Molecular Medicine, Queen's University, Kingston, ON K7L3N6, Canada

**Keywords:** Hippo, TAZ (WWTR1), Cdk1, antitubulin drug, chemoresistance

## Abstract

Antitubulin drugs are commonly used for the treatment of numerous cancers. However, either the intrinsic or acquired resistances of patients to these drugs result in the failure of the treatment and high mortality of cancers. Therefore, identifying genes or signalling pathways involved in antitubulin drug resistances is critical for future successful treatment of cancers.

TAZ (Transcriptional coactivator with PDZ-binding motif), which is a core component of the Hippo pathway, is overexpressed in various cancers. We have recently shown that high levels of TAZ in cancer cells result in Taxol resistance through up-regulation of downstream targets Cyr61 and CTGF. However, how TAZ is regulated in response to Taxol is largely unknown. In this study, we found that Cdk1 (Cyclin-dependent kinase 1) directly phosphorylated TAZ on six novel sites independent of the Hippo pathway, which further resulted in TAZ degradation through proteasome system. Phosphorylation-mimicking TAZ mutant was unstable, and therefore abolished TAZ-induced antitubulin drug resistances. This study provides first evidence that Cdk1 is a novel kinase phosphorylating and regulating TAZ stability and suggests that Cdk1-TAZ signalling is a critical regulator of antitubulin drug response in cancer cells and may be a potential target for the treatment of antitubulin-drug resistant cancer patients.

## INTRODUCTION

Antitubulin drugs including paclitaxel (Taxol) and vinblastine are the chemotherapeutic drugs widely used for clinical cancer treatment of several types of cancers such as breast cancer, non-small cell lung cancer (NSCLC), ovarian cancer, etc [1–4]. However, cancer patients either have intrinsic resistance to these drugs or gradually develop acquired resistance during chemotherapy, which results in the failure of chemotherapy and high mortality [5, 6]. Normally the resistance of certain drugs depends on the molecular alterations of cancer cells. Therefore, studying the molecular mechanisms underlying the responses of cancer cells to chemotherapeutic drugs may provide useful information in dealing with chemoresistance.

TAZ (Transcriptional co-activator with PDZ binding motif), also named as WWTR1 (WW domain containing transcription regulator 1), is one of the core components (MST-LATS-YAP/TAZ) in the Hippo pathway that plays important roles in tumorigenesis, organ size control, stem cell differentiation and renewal, etc [7–11]. It is well-known that TAZ can be phosphorylated by LATS (Large tumor suppressor) through four serine (S) sites, which can cause TAZ either bind to 14–3–3 protein and is anchored in cytoplasm or be degraded through ubiquitin-proteasome-system (UPS) by interacting with E3 ubiquitin ligase β-TRCP [12, 13]. High expression of TAZ is found in several types of cancers and several studies suggest that TAZ is related to the malignancy of breast cancer and lung cancer as well as cancer stem cell property maintenance and chemoresistance [14–19]. Our lab previously identified that TAZ upregulates its downstream targets, Cyr61(cysteine rich, angiogenic 61) and CTGF (connective tissue growth factor), through interacting with TEAD (TEAD domain)/TEF (transcriptional enhancer factor) transcription factor, which further leads to the resistance of breast cancer cells to antitubulin drug Taxol [14]. However, the upstream genes regulating TAZ-induced sensitivity to antitubulin drugs are largely unknown. Here in this study, we found that TAZ was phosphorylated and degraded in Taxol sensitive cells during Taxol treatment. The phosphorylation of TAZ was due to activated Cdk1 on six novel sites of TAZ, which subsequently led to TAZ degradation and therefore hindered the involvement of TAZ in drug resistance. This study discovers an essential role of Cdk1-TAZ signalling in determining the tumor cell sensitivity to antitubulin drugs and suggests a novel signalling target for the treatment of antitubulin drug-resistant cancers.

## RESULTS

### TAZ is phosphorylated and degraded during Taxol treatment

**Figure 1:**
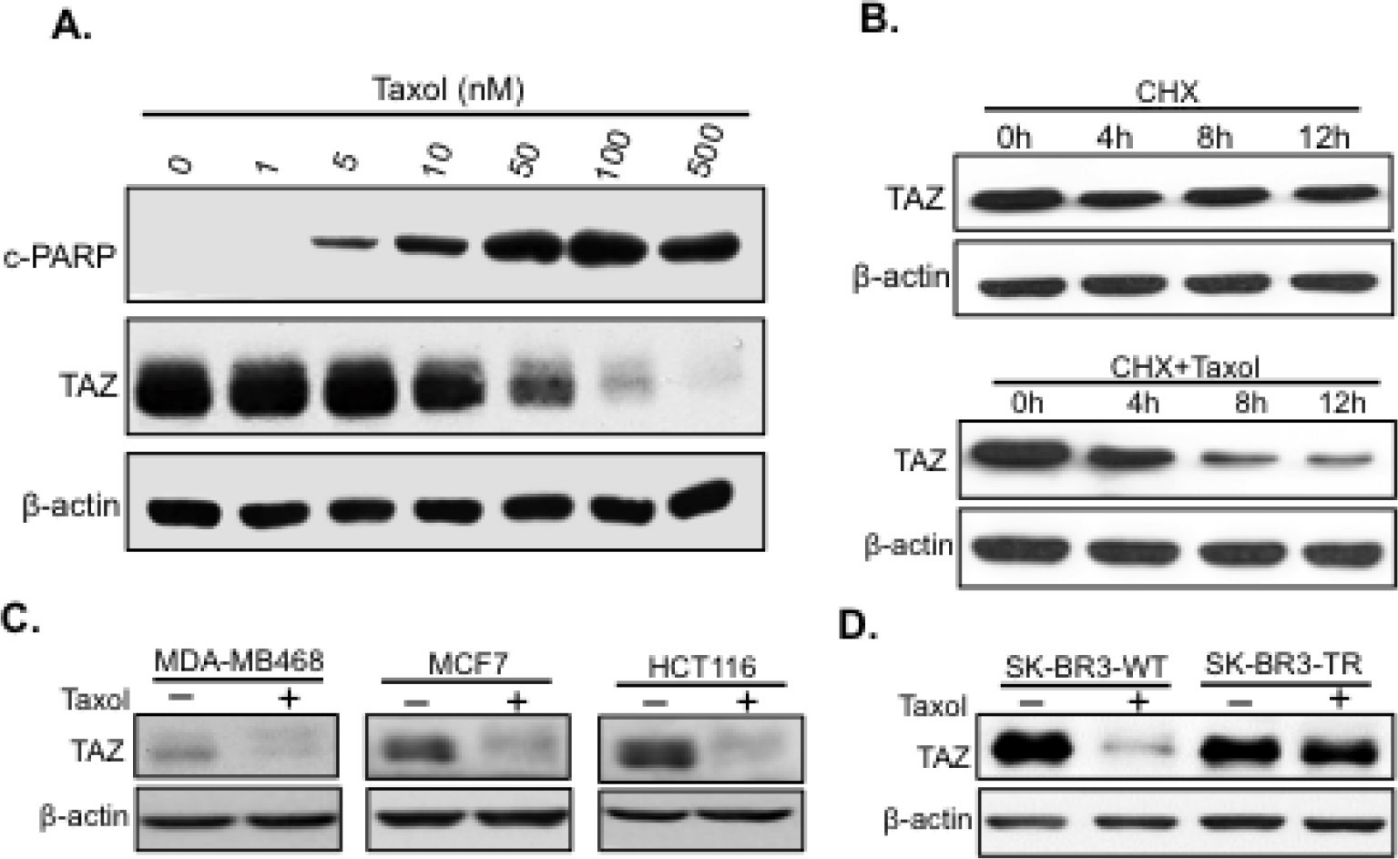
Taxol causes TAZ phosphorylation and degradation **A.** HeLa cells were treated with Taxol at different concentrations (0, 1, 5, 10, 50, 100, or 500 nM) for 24 hours before cells were lysed for western blot to detect the apoptotic marker, cleaved-PARP (cPARP), and TAZ with related antibodies. β-actin was used as an internal loading control. **B.** Western blot analysis of TAZ turnover time after Taxol treatment. HeLa cells were treated with cycloheximide (CHX, 60 μg/ml) to block TAZ protein synthesis in the absence (−) or presence (+) of Taxol (100 nM). Cells were lysed at 0, 4, 8, or 12 h after drug treatments and subjected to western blot analysis of TAZ. **C.** TAZ is also phosphorylated and degraded in other cell lines. Breast cancer cell lines, MDA-MB468 and MCF7 as well as colon cancer cells HCT116 were treated with Taxol at 100 nM for 24 hours and floating cells were collected and lysed for western blotting to detect TAZ protein phosphorylation and degradation. **D.** Loss of TAZ degradation after Taxol treatment in drug resistant SK-BR3-TR cells. Cells were treated as described above. Wild-type SK-BR3 (SK-BR3-WT) as well as Taxol-resistant SK-BR3 cells (SK-BR3-TR) were cultured in the absence or presence of Taxol for 24 hours and TAZ levels were examined by western blot.

To examine how TAZ responds to Taxol treatment, TAZ levels were examined in HeLa cells treated with Taxol at different dosage for 24 hours. TAZ was found phosphorylated and degraded with the increase of Taxol dosage and apoptosis indicated by cleaved PARP (cPARP), an apoptotic marker (Figure 1A). To test whether reduced levels of TAZ is due to increased TAZ turnover after Taxol treatment, we treated cells with cycloheximide (CHX) to inhibit protein synthesis in the absence or presence of Taxol. Significantly, compared to cells treated only with CHX, Taxol treatment together with CHX caused increased TAZ degradation (Figure 1B), suggesting that Taxol reduces TAZ levels by causing TAZ protein degradation. Besides, TAZ was phosphorylated and degraded in different types of cancer cells with Taxol treatment (Figure 1C), suggesting this Taxol-induced TAZ degradation is not cell line specific. Moreover, TAZ is degraded in wild-type (WT) rather than Taxol-resistant (TR) SK-BR3 breast cancer cells (Figure 1D). Together, these findings strongly suggest that degradation of TAZ is correlated with the sensitivity of cancer cells to antitubulin drug Taxol.

### TAZ degradation after taxol treatment is not due to the taxol-induced cell detachment or the activation of the Hippo pathway

**Figure 2:**
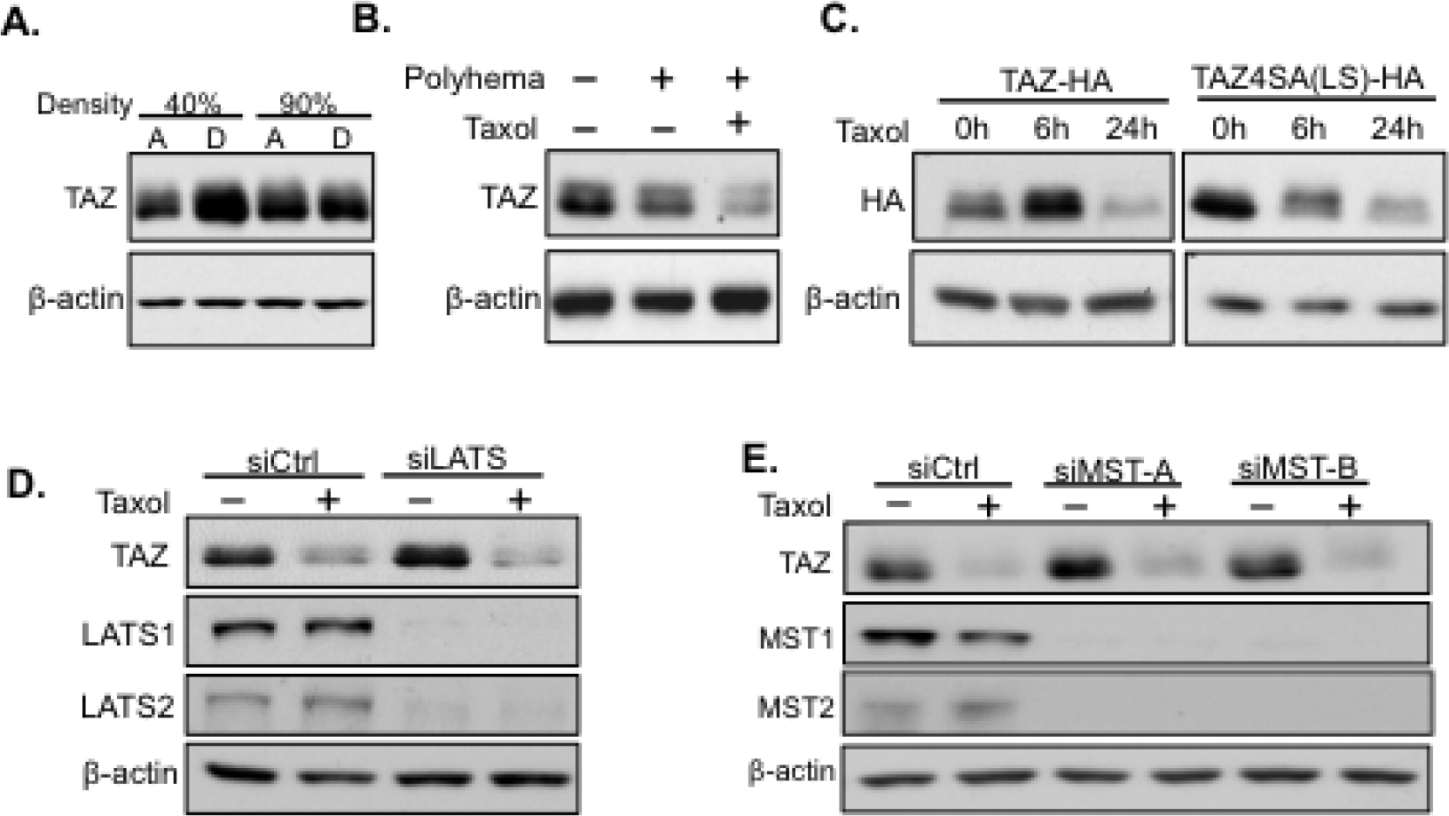
Taxol-induced TAZ degradation is independent of the Hippo pathway and cell detachment or floating **A.** TAZ is not degraded due to cell detachment. HeLa cells were trypsinized and lysed (D) or directly lysed (A) at either 40% or 90% confluency to mimic cell detachment (D) and attachment (A) Then cell lysates were subjected to western blot to detect TAZ expression. **B.** TAZ is not degraded due to cell floating. To check TAZ expression in floating conditions, HeLa cells were cultured in poly-hema coated plates which blocked cell attachment. Taxol (100 nM) were added to one plate of cells at the right start of floating culture. Cells either cultured in floating condition alone or together with Taxol treatment in floating culture were collected after 24 hours and lysed for western blot to detect TAZ expression with cells without floating culture as a control. **C.** Taxol can still cause degradation of TAZ mutant with LATS1/2 phosphorylation site mutations [TAZ4SA (LS)]. HA-tagged wild-type TAZ or TAZ4SA (LS) was transfected into HeLa cells and subsequently treated with Taxol (100 nM) for either 6 hours or 24 hours. Floating cells were collected and lysed for western blot analysis of TAZ expression using anti-HA antibody. **D, E.** Taxol induced TAZ degradation is independent of the upstream Hippo pathway. The siRNAs targeting the Hippo pathway core components, LATS1/2 (siLATS) (D) or MST1/2 (siMST-A/B) (E), were introduced into HeLa cells, followed by untreated or treated with Taxol (100 nM) for 24 h. Cells were lysed for western blot to detect TAZ expression with MST1/2 and LATS1/2 expression as controls for checking the efficiency of siRNA knocking down.

Cells are usually detached from the cell culture plate and remain in floating condition after Taxol treatment. To exclude the possibility that TAZ degradation is due to the detachment of cells instead of Taxol related regulation, HeLa cells were trypsinized at both low cell density (40%) and high cell density (90%). However, TAZ was neither degraded nor phosphorylated after detachment (Figure 2A). To test if the floating condition plays a role in Taxol-induced TAZ alteration, HeLa cells were cultured in Polyhema-coated plates to mimic the floating condition. Cells were collected after 24 hours culture with or without Taxol (100 nM) added. The expression level of TAZ was only significantly decreased in floating cells treated with Taxol (Figure 2B). Together these experiments indicate that TAZ alteration was directly through Taxol treatment rather than Taxol-induced cell detachment or floating.

It is well known that TAZ is directly regulated by LATS kinase in the Hippo pathway (**MST-LATS-TAZ**). LATS phosphorylates four “S” sites (S66, S89, S117, S311) in TAZ, among which, S311 phosphorylation induces TAZ degradation, while S89 phosphorylation can anchor TAZ in the cytoplasm through interaction with 14–3–3 protein [12, 13]. To detect if Taxol induces TAZ phosphorylation and degradation through LATS, we first transfected WT TAZ-HA or TAZ mutant, which has all the four LATS (LS) phosphorylation sites mutated from “S” to Alanine (A)[TAZ4SA (LS)], followed by Taxol treatment for 0, 6, and 24 h. Surprisingly, both TAZ-HA and TAZ-4SA(LS)-HA were degraded after Taxol treatment for 24 hours (Figure 2C), suggesting that phosphorylation of TAZ by LATS is not essential for Taxol-induced TAZ degradation. Interestingly, TAZ levels were enhanced after 6 h Taxol treatment. Since Taxol induces mitotic arrest during early stage of treatment, it is possible that TAZ is activated to counter Taxol-induced mitotic cell cycle arrest. To exclude the possibility that Taxol may activate LATS to phosphorylate TAZ on sites other than the known four LATS phosphorylation sites (4S) to cause degradation, LATS1/2 (LATS) was knocked down by siRNAs in HeLa cells, followed by Taxol treatment. However, TAZ was still phosphorylated and degraded after LATS knockdown (Figure 2D). Similar results were obtained when LATS upstream kinase, MST1/2 (mammalian Hippo), were knocked down by siRNAs, followed by Taxol treatment (Figure 2E). Together, these results highly suggest Taxol-induced degradation of TAZ is independent of the Hippo pathway.

### Taxol induces TAZ phosphorylation and degradation through Cdk1

**Figure 3:**
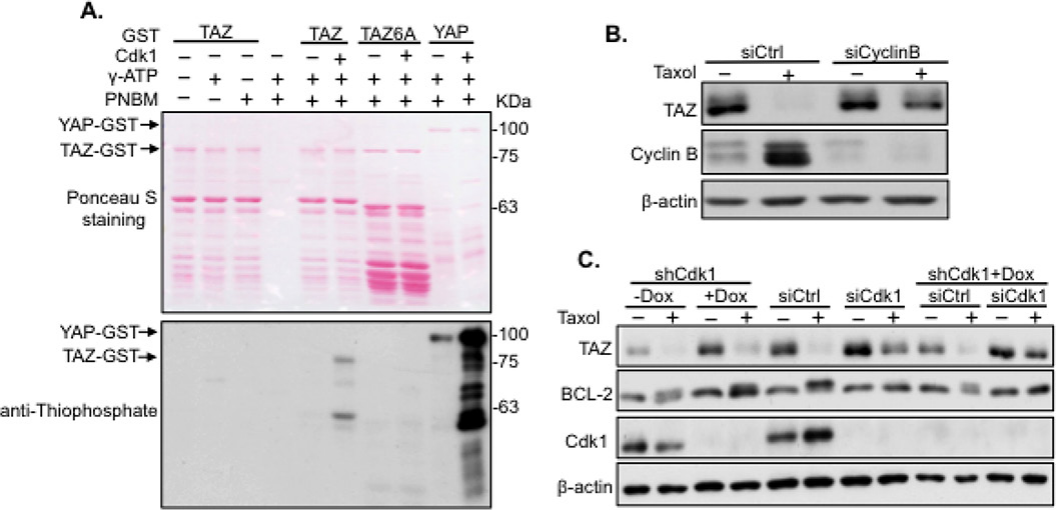
Taxol-induced TAZ phosphorylation and degradation is mediated by Cdk1 **A.** Phosphorylation of TAZ by Cdk1 *In vitro*. TAZ-GST or TAZ6A-GST was incubated with Cdk1 kinase, followed by *in vitro* kinase assay. YAP-GST was used as a positive control for Cdk1 kinase activity. Upper panel: ponceau S staining of fusion proteins on the membrane; Lower panel: western blot analysis of phosphorylated TAZ using SDS-PAGE gel and anti-thiophosphate antibody. **B.** Knockdown of Cyclin B blocks Taxol-induced TAZ degradation. Cyclin B was knocked down by siRNA in HeLa cells in the absence (−) or presence (+) of Taxol (100 nM) for 24 hours. Cells were lysed for western blot to detect TAZ expression. **C.** Knockdown of Cdk1 abolishes Taxol-induced Cdk1 degradation. Cdk1 was targeted with siCdk1, shCdk1 [inducible system with Doxycyclin (Dox) to induce shCdk1] and the combination of siCdk1 and shCdk1 in HeLa cells, and treated with Taxol (100 nM) for 24 hours. Cells were collected and lysed for western blot to detect TAZ expression. Since BCL-2 is a known substrate of Cdk1, BCL-2 was used as a control to check the efficiency of Cdk1 knocking down.

**Figure 4:**
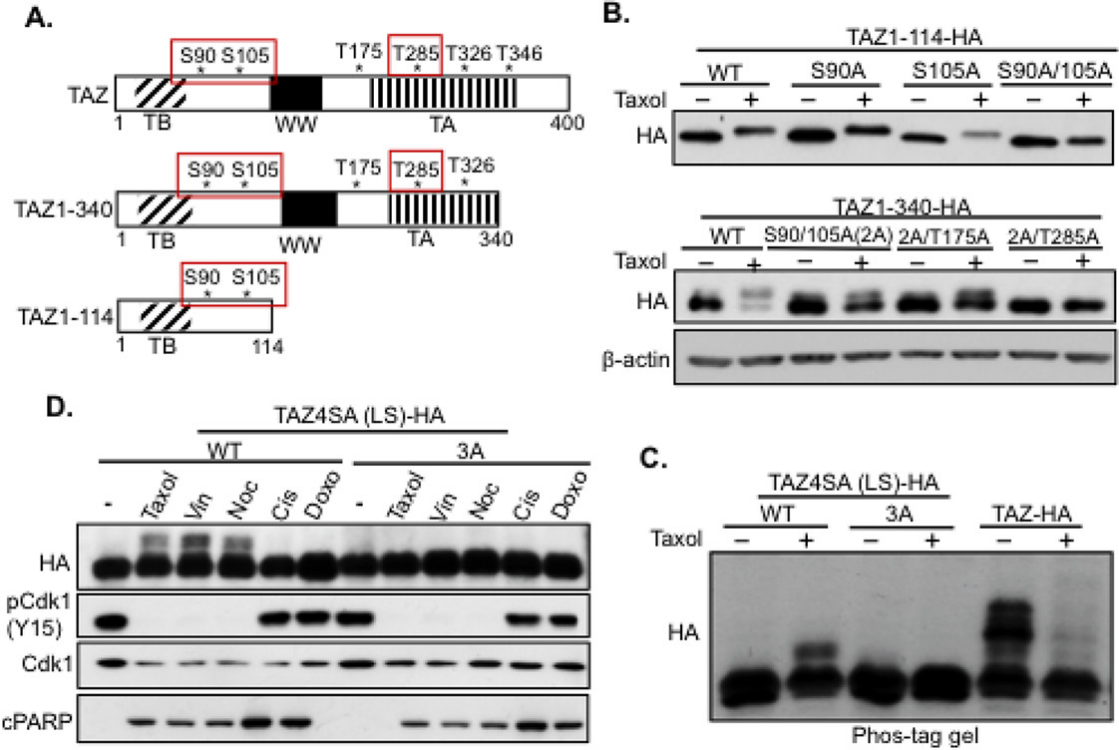
Mapping the Taxol-induced Cdk1 phosphorylation sites in TAZ **A.** Schematic diagraph of TAZ functional domains and potential phosphorylation sites. TB, TEAD binding domain; WW, WW domain; TA, transactivating domain. S90, S105, and T285 are highlighted to indicate they are the phosphorylation sites causing band-shift after Taxol treatment for 6 h. **B.** Deletion and mutation analysis of TAZ phosphorylation. TAZ 1–114 and TAZ 1–340 deletions were used as templates to mutate the potential “S/T” phosphorylation sites into “A” singly or in combination. And these constructs were transfected into HeLa cells with Taxol treatment for 6 h to detect the sites responsible for Taxol-induced TAZ phosphorylation (band-shift on the gel) through running the floating (dead) cell lysates on western blot. **C.** Confirmation of Cdk1 phosphorylation sites by phos-tag gel. The three phosphorylation sites (S90, S105, and T285) identified with TAZ deletions (A, B) were further mutated into “A” (3A) on full length TAZ4SA (LS) (all 4 LATS phosphorylation sites are mutated). And this mutant [TAZ4SA (LS)+TAZ3A], TAZ4SA (LS) (WT) or wild-type TAZ was transfected into HeLa cells and treated with Taxol for 6 h. Then the lysates of floating cells were subjected to phos-tag gel to detect the potential extra phosphorylation sites, which only shift on phos-tag gel. Please note that phos-tag gel detects many LATS-phosphorylated TAZ proteins even without Taxol treatment. **D.** TAZ phosphorylation is specifically induced by antitubulin drugs. TAZ4SA (LS) (LATS phosphorylation site mutant) or TAZ4SA (LS)+3A (LATS phosphorylation site mutants plus 3A) were transfected into HeLa cells and treated with antitubulin drugs [Taxol, Vin (Vinblastine), Noc (Nocodazole)] at 100 nM as well as DNA damage reagents [20 μM Cis (Cisplatin) and 1 uM Doxo (Doxorubucin)] for 6 h. Phosphorylation of TAZ was detected with phos-tag gel, and Cdk1 activity was detected by checking phosphorylation on Y15 of Cdk1 (pCdk1-Y15). Apoptosis was determined by detecting cPARP expression.

In our previous work, YAP was phosphorylated by Cdk1 on five phosphorylation sites during Taxol treatment [20]. Since TAZ is the paralog of YAP, it is possible Taxol-induced TAZ alteration is Cdk1 dependent. Cdk1 specifically recognizes “S” or Threonine (T) in a “SP/TP” (P, Proline) motif [21]. By examining potential Cdk1 phosphorylation sites in TAZ, 6 S/T sites (S90, S105, T175, T285, T326, and T346) with S/TP motifs were identified (Figure 4A). To check if TAZ can be phosphorylated by Cdk1, *in vitro* kinase assay with Cdk1 kinase was performed using TAZ-GST or TAZ6A-GST (all 6 potential S/T Cdk1 phosphorylation sites were mutated into A, Figure 4A) fusion protein as the substrate and YAP-GST protein as a positive control. TAZ rather than TAZ6A was phosphorylated when Cdk1 was added (Figure 3A), suggesting Cdk1 can directly phosphorylate TAZ. Furthermore, siRNA targeting Cyclin B, the functional partner of Cdk1, was used together with Taxol treatment. Taxol-induced TAZ degradation was blocked after knocking down Cyclin B (Figure 3B). Moreover, siRNA/shRNAs targeting Cdk1 were introduced into HeLa cells, followed by Taxol treatment. Like BCL-2 positive control, TAZ was found no longer phosphorylated and degraded in cells with Cdk1 completely knocked down by the combination of siCdk1 and shCdk1 (Figure 3C), further confirming that Cdk1 is the kinase regulating TAZ during Taxol treatment.

To narrow down the actual Cdk1 phosphorylation sites, two TAZ deletions (TAZ1–114 and TAZ1–340) containing part of the potential phosphorylation sites mutated into A (SA or TA) were constructed and transfected into HeLa cells, followed by treatment with Taxol for 6 h at which time point TAZ is significantly phosphorylated but less degraded (Figure 2C). Western blot analysis shows that three phosphorylation sites (S90, S105, and T285) contribute to the band-shift (phosphorylation) of TAZ protein after Taxol treatment (Figure 4B). To further confirm whether these three sites are the only major sites for Taxol-induced TAZ phosphorylation, a phos-tag gel was used to identify any phosphorylation site that does not cause band-shift on regular SDS-PAGE gel. Since phosphorylation of TAZ by endogenous LATS also causes multiple bands of wild type TAZ on phos-tag gel even without Taxol treatment (Figure 4C), TAZ4SA (LS), which mutates all four LATS “S” phosphorylation sites into “A”, was used as a template to further have the Taxol-induced three Cdk1 phosphorylation sites (S90, S105, and T285) mutated into “A” [TAZ4SA (LS)+3A]. “Wild-type” (WT) TAZ4SA (LS) had a band-shift after 6 h of Taxol treatment (Figure 4C), indicating that Taxol-induced phosphorylation of TAZ is independent of the four LATS phosphorylation sites. However, the band-shift of TAZ4SA (LS) was abolished by mutating the three potential Taxol-induced Cdk1 phosphorylation sites [TAZ4SA (LS)+3A], verifying that these three sites are fully responsible for Taxol-induced TAZ phosphorylation by Cdk1 (Figure 4C). To examine if this TAZ regulation is specific to Taxol, both TAZ4SA (LS) and TAZ4SA (LS)+3A were treated with three different antitubulin drugs (Taxol, Vinblastine, or Nocodazole), as well as two DNA damage drugs (Cisplatin or Doxorubicin) for 6 h. Then phosphorylations of TAZ with different drug treatments were detected through running the cell lysates on phos-tag gel (Figure 4D). Interestingly, TAZ4SA (LS)(WT) was phosphorylated specifically after antitubulin drug treatments and this phosphorylation was abolished on TAZ4SA (LS)+3A (Figure 4D), suggesting that this Cdk1-induced phosphorylation of TAZ is antitubulin drug specific.

**Figure 5:**
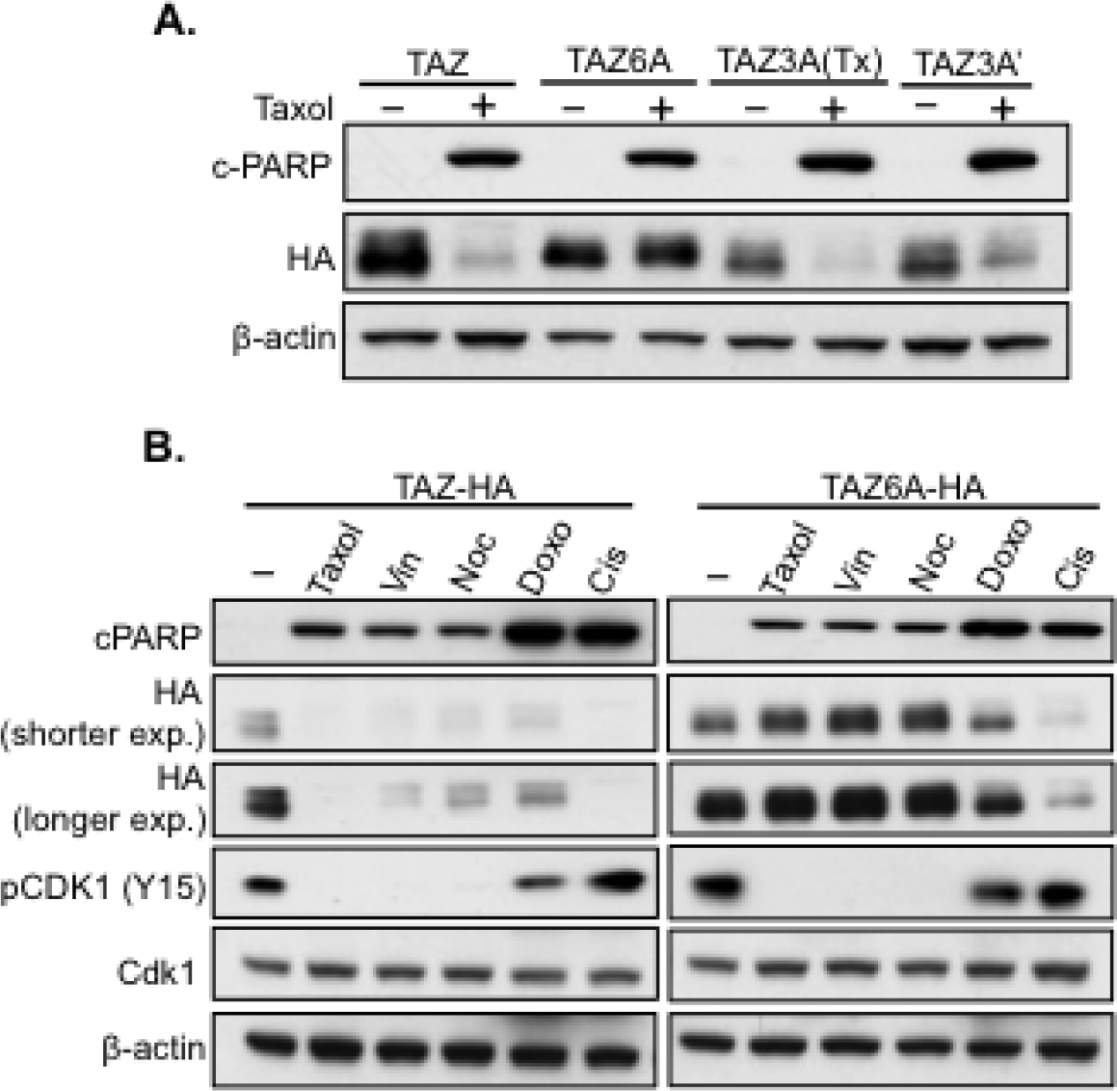
TAZ degradation after antitubulin drug treatment depends on six phosphorylation sites **A.** Phosphorylation of six S/T sites is essential for Taxol-induced TAZ degradation. TAZ, TAZ3A (S90A/S105A/T285A), TAZ3A’ (T175/T326A/T346A) or TAZ6A (3A+3A′) were cloned into HA-tagged vector and transfected into HeLa cells and treated with Taxol (100 nM) for 24 h, followed by western blot analysis of cPARP and TAZ. **B.** Mutations of Cdk1 phosphorylation in TAZ only block antitubulin drug-induced TAZ degradation. TAZ-HA or TAZ6A-HA was overexpressed in HeLa cells and treated with 100 nM of antitubulin drugs (Taxol, Vin, Noc), 20 μM of Cis or 1 μM of Doxo for 24 h. The stability of TAZ or TAZ6A, Cdk1 activity (pCdk1-Y15) and apoptosis (cPARP) were examined by western blot.

Unlike YAP during antitubulin drug treatments [20], TAZ is not only phosphorylated but also degraded (Figure 1A). But when Cdk1 was knocked down, TAZ was no longer degraded (Figure 3C), suggesting Cdk1 is also responsible for Taxol-induced TAZ degradation. Therefore, we started to examine if the three Cdk1 phosphorylation sites identified were responsible for TAZ degradation as well. Therefore, TAZ3A construct, which has three identified Taxol-induced phosphorylation sites mutated, was transfected into HeLa cells and treated with Taxol for 24 hours, followed by western blot analysis of TAZ levels. To our surprise, both TAZ3A (S90, S105 and T285 are mutated into “A”) and TAZ3A’ [T175, T326 and T346 mutated into “A” (Figure 4A)] were still degraded after Taxol treatment (Figure 5A). Interestingly, when all 6 potential Cdk1 phosphorylation sites were mutated from “S” into “A” (TAZ6A), TAZ degradation was completely abolished (Figure 5A), indicating that all the six phosphorylation sites are indispensable for Cdk1-regulated TAZ stability. To exclude the possibility that resistance to Taxol-induced degradation in TAZ6A is due to protein conformation changes caused by mutations themselves other than the blockage of Cdk1 phosphorylation, expressions of TAZ6A and TAZ were examined after transfection of these constructs into HeLa cells and treated with various drugs for 24 hours. TAZ6A was only stable after treatment of antitubulin drugs but not DNA-damage related drugs (Figure 5B), which confirmed that these six phosphorylation sites in TAZ are Cdk1-specific and are responsible for antitubulin drug-induced TAZ degradation. However, only three sites were identified after 6 h of Taxol treatment (Figure 4A–4B), and this three site mutant (TAZ3A) could not block TAZ degradation after 24 h of Taxol treatment (Figure 5A). Since TAZ is dramatically degraded with Taxol treatment for 24 hours instead of 6 hours (Figure 2C), it is possible that the rest three sites were phosphorylated after longer Taxol treatment. To explore this possibility, TAZ4SA (LS) and TAZ4SA (LS)+3A were treated with Taxol for 8 hours, 12 hours and 18 hours, followed by phos-tag gel analysis. As expected, extra phosphorylation (shifted band) of TAZ4SA (LS)+3A was identified with longer (12 h or 18 h) Taxol treatment (Supplementary Figure 1). It is possible that with longer drug treatments, Cdk1 will get more activated and as a result, more sites on TAZ will be phosphorylated, which finally cause TAZ degradation. In conclusion, these studies strongly suggest that Taxol induces TAZ phosphorylation and degradation through phosphorylation of six S/T sites by Cdk1.

### Taxol induces TAZ degradation through ubiquitin-proteasome-system (UPS)

**Figure 6:**
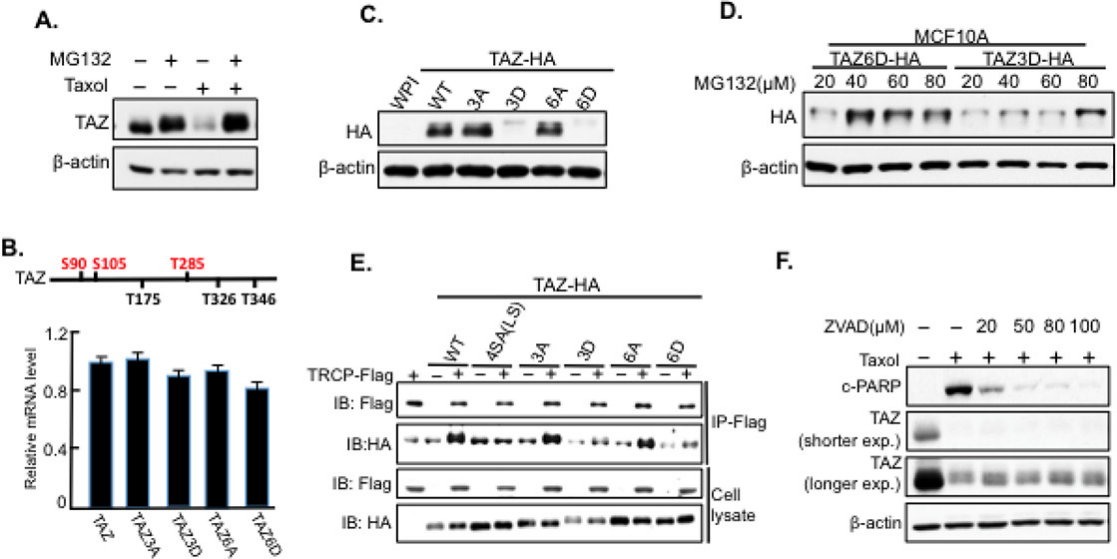
TAZ is degraded through the proteasome system independent of β-TRCP **A.** MG132 blocks Taxol-induced TAZ degradation. HeLa cells treated with MG132 (20 μM) singly or together with Taxol (100 nM) for 24 h before TAZ was detected by western blot. **B.** Equal levels of mRNAs for TAZ and its mutants in MCF10A cells. MCF10A cells stably overexpressing different TAZ mutants [TAZ, TAZ3A, TAZ3D, TAZ6A, and TAZ6D] were established through lentiviral infection. And RNA was extracted from each infected cell lines for qRT-PCR to detect TAZ mRNA levels in each cell line. **C.** Phosphorylation-mimicking TAZ mutants are unstable. Each stable cell line with similar TAZ mRNA expressions was lysed for western blot to detect the protein levels of TAZ. **D.** MG132 treatment restores the stability of phosphorylation-mimicking TAZ. MG132 was used to treat MCF10A stably overexpressing the phosphorylation-mimicking mutants TAZ3D and TAZ6D at four different concentrations (20, 40, 60, 80 μM) for 8 hours before cells were lysed for western blot. **E.** Interaction of TAZ and β-TRCP is independent of TAZ phosphorylation. Co-IP was performed by transfecting HA-tagged TAZ or its mutant alone (−) or together with β-TRCP-Flag (+) into HeLa cells. Two days after co-transfection, cells were treated with MG132 for 8 hours prior to protein extractions. Immunoprecipitation was performed with Flag (M2) antibody to pull down β-TRCP-Flag protein, and different TAZ mutants were detected in the pull down complexes by HA antibody. **F.** Taxol-induced TAZ degradation is not the result of apoptosis. HeLa cells were treated with pan-caspase inhibitor (ZVAD) at different concentrations (0, 20, 50, 80, 100 μM) in the absence (−) or presence of Taxol (100 nM) for 24 hours, followed by protein extraction and western blot analysis. cPARP was used as the marker for apoptosis.

In present study, we found that TAZ is degraded through Cdk1 in antitubulin drug sensitive cells. Most intracellular proteins are targeted for degradation through UPS [22]. Thus, we next examined weather TAZ degradation was through proteasome. By co-treating HeLa cells with Taxol together with MG132, a proteasome inhibitor, Taxol-induced TAZ degradation was inhibited (Figure 6A), revealing TAZ degradation is through proteasome system. In addition, we established Taxol-sensitive MCF10A (immortalized mammary epithelial cells) cell lines stably overexpressing different TAZ mutants [phosphorylation site inactivating mutants, TAZ3A and TAZ6A; phosphorylation mimicking mutants, TAZ3D and TAZ6D (D, aspartic acid)] through lentiviral infection system. Each stable cell line with equal mRNA levels of TAZ was selected according to qRT-PCR analysis (Figure 6B). Both TAZ3A and TAZ6A are as stable as wild-type TAZ (Figure 6C). However, the phosphorylation mimicking mutants, TAZ3D and TAZ6D, had dramatic decreased protein expressions (Figure 6C). And these decreased expressions of TAZ3D and TAZ6D were inhibited with MG132 treatments (Figure 6D), confirming that phosphorylations of TAZ by Cdk1 indeed causes TAZ instability through proteasome system. Interestingly, while mutations of all the six sites (TAZ6A) are required to block Taxol-induced TAZ degradation, TAZ3D, which mimics phosphorylation of three sites (S90/S105/T285) in TAZ in the early stage of antitubulin drug treatments, is sufficient to cause TAZ instability (Figure 5A, 6C). To elucidate the roles of other three Cdk1 phosphorylation sites (T175/T326/T346, Figure 4A) in Taxol-induced TAZ degradation, we also made another two constructs: TAZ3A-3D’, which further mimic the phosphorylation of the rest three sites on TAZ3A, and TAZ3D-3A’, which abolish the phosphorylation of the rest three sites on phosphorylation mimicking TAZ3D (Supplementary Figure 2A). Western blot analysis of TAZ shows that TAZ3A-3D’ is stable while TAZ3D-3A’ significantly inhibits TAZ degradation (Supplementary Figure 2B), suggesting that phosphorylation of other three sites (T175/T326/T346) is required but not sufficient for Taxol-induced TAZ degradation.

In general, protein targeted to be degraded by proteasome needs to be tagged by ubiquitin first [23, 24]. And this specific targeting process depends on the interactions between the protein and the E3 ubiquitin ligases. Several studies indicate that, the F-box family protein, β-TRCP, can interact with and cause ubiquitinization and degradation of TAZ [12, 25–27]. Besides, β-TRCP is also involved in Cdk1 regulated Wee1 degradation [28], suggesting a potential role of β-TRCP in Cdk1-regulated TAZ degradation. To check if β-TRCP is involved in Taxol-induced TAZ degradation, we examined the interaction of β-TRCP with wild-type TAZ (TAZ-WT) and its mutants [TAZ4SA (LS), TAZ3A, TAZ3D, TAZ6A, or TAZ6D] through co-immunoprecipitation (co-IP). TAZ4SA (LS) was used as negative control since it is well known that LATS phosphorylation on TAZ promotes the interaction between β-TRCP and TAZ, resulting in TAZ degradation [12]. As expected, although TAZ-WT interacts with β-TRCP strongly, TAZ4SA(LS) significantly reduces its binding to β-TRCP (Figure 6E). However, no increased interactions between β-TRCP and the two phosphorylation mimicking mutants (TAZ3D and TAZ6D) were identified (Figure 6E), indicating that Taxol-induced TAZ degradation is independent of β-TRCP.

Since TAZ degradation is correlated with Taxol-induced apoptosis (Figure 1A), we investigated the relation between TAZ degradation and apoptosis. It is well-known that apoptosis results from activation of caspases [29]. Therefore, a pan-caspase/apoptosis inhibitor (Z-VAD) was used at different concentrations to treat cells together with Taxol. TAZ was still degraded when apoptosis was inhibited (Figure 6F), indicating that TAZ degradation is the cause (upstream mediator) rather than the result of Taxol-induced apoptosis.

### TAZ phosphorylation by Cdk1 sensitizes cells to antitubulin drugs

**Figure 7:**
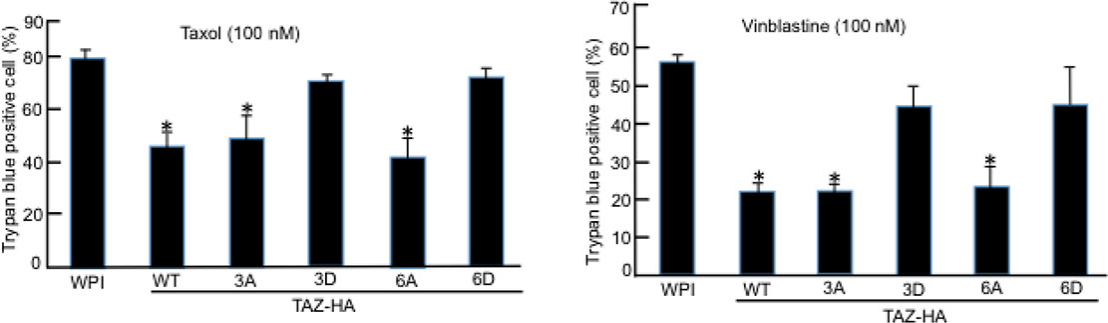
TAZ phosphorylation by Cdk1 sensitizes cells to antitubulin drugs MCF10A cells stably expressing WPI vector, TAZ, or TAZ mutants (TAZ3A, TAZ3D, TAZ6A, or TAZ6D) were cultured in triplicate 12-well plate at 40% confluency before adding Taxol or Vinblastin at concentration of 100 nM. Cells were cultured for 48 hours with drugs added before subjected to Trypan blue exclusion assay. The experiments were repeated for three times and the average data and standard errors were calculated. * indicates the difference between WPI control and TAZ-WT or TAZ-3A or TAZ-6A mutants is statistically significant (*p* < 0.05).

Since TAZ overexpression in MCF10A cells can result in their resistance to Taxol [14], we next examined the roles of TAZ phosphorylation by Cdk1 by treating MCF10A cells stably overexpressing TAZ or different TAZ phosphorylation mutants (TAZ, TAZ3A, TAZ3D, TAZ6A, or TAZ6D) with antitubulin drugs, Taxol and Vinblastine for 48 hours. Similar to wild-type TAZ, overexpression of TAZ3A or TAZ6A in MCF10A cells caused resistant to both Taxol and Vinblastine (Figure 7). On the other hand, overexpression of TAZ with phosphorylation mimicking mutations (TAZ3D and TAZ6D) had only minimum effects on MCF10A cells in response to antitubulin drugs (Figure 7), further confirming that phosphorylation of TAZ by Cdk1 abolishes its anti-apoptotic function in drug resistance.

## DISCUSSION

TAZ is one of the core components of the Hippo pathway that is involved in various physiological and pathological activities, such as organ size control, tumorigenesis, tissue homeostasis, energy homeostasis [8–10, 30–33]. We and others have recently shown that dysregulation of the core Hippo pathway components such as LATS, YAP, and TAZ results in resistances of tumors to various chemotherapeutic drugs such as Taxol, Doxorubicin, erlotinib, RAF/MEK inhibitors, etc [19, 34]. However, how the Hippo pathway is involved in chemotherapeutic drug responses is still not fully understood. Here in this study we found that TAZ is regulated through Cdk1 rather than the canonical Hippo pathway. Phosphorylation of TAZ by Cdk1 further induces TAZ degradation through ubiquitin-proteasome system, which therefore eliminates TAZ's functions in anti-apoptosis and cell resistance to antitubulin drugs.

It is well-known that Cdk1 is the major kinase essential for the progression of cell cycle from G_2_ phase into mitosis during cell proliferation. Since TAZ is an oncogene and overexpression of TAZ increases cell proliferation [13], therefore, it is also possible that Cdk1 may phosphorylate and activate TAZ to promote mitotic progression during cell proliferation. However, it has also been shown that in response to antitubulin drugs, Cdk1 kinase is dramatically activated, which can trigger apoptosis [35, 36]. Therefore, cells with highly activated Cdk1 are more sensitive to antitubulin drug treatments, while Cdk1 inhibitors can protect cancer cells from antitubulin-induced apoptosis [35, 36]. However, why and how Cdk1 activity determines the sensitivity of cells to antitubulin drugs is not fully understood. Our studies suggest that phosphorylation and degradation of TAZ by Cdk1 may play important roles in apoptosis induced by antitubulin drugs. Under physiological conditions, Cdk1 activity is low. Therefore, both TAZ and other downstream Cdk1 targets could promote cell cycle progression and cell proliferation. However, when Cdk1 is activated by antitubulin drugs, it can lead to significant TAZ phosphorylation and subsequent UPS-mediated degradation, resulting in apoptotic cell death. On the other hand, when TAZ is overexpressed in tumor cells, there are not enough Cdk1 molecules in cells to phosphorylate and inactivate TAZ, which will cause resistance of tumor cells to antitubulin drug-induced apoptosis. Therefore, the Cdk1-TAZ signalling axis may be a critical sensor in determining cell proliferation or cell death under physiological and stress (microtubule damage by antitubulin drugs) conditions.

In our previous study, Cdk1 was identified to contribute to antitubulin drugs-induced apoptosis by phosphorylating YAP (Yes-associate protein), a paralog of TAZ, and inhibiting its transactivating function [20]. We showed that Cdk1 phosphorylation on YAP decreases the interaction between YAP and the transcriptional factor TEAD, which therefore blocks the expressions of downstream target genes (e.g., *Cyr61* and *CTGF*) involved in Taxol resistance [20]. In this study, we found Cdk1 can inactivate TAZ by a different mechanism. We have provided convincing evidence that after Taxol treatment Cdk1 can first phosphorylate TAZ on three sites (S90A/S105/T285), which causes further phosphorylation on other three sites (T175/T326/T346) and subsequently leads to proteasome-mediated degradation. Although TAZ3A (S90A/S105A/T285A) was degraded at similar rate to TAZ-WT and TAZ3A′ (T175A/T326A/T346A) was still partially degraded, TAZ6A was stable after Taxol treatment, suggesting that phosphorylation of all 6 sites is essential for Taxol-induced TAZ degradation. The molecular mechanism underlying this differential regulation of YAP and TAZ by Cdk1 is unknown. However, by comparing the Cdk1 phosphorylation sites in YAP (S128, S138, S217, S289, and S367) and TAZ (S90, S105, T175, T285, T326, and T346), we found that there are only two Cdk1 phosphorylation sites (TAZ: S90, T326; YAP: S128 and T412) that are conserved. Since phosphorylation of six sites in TAZ by Cdk1 is required for Taxol-induced TAZ degradation (Figure 5, Supplementary Figure 2), it is possible that YAP contains only two conserved phosphorylation sites, which are not sufficient to cause proteasome-induced degradation. Besides, compare to YAP, TAZ has one extra degron, which can interact with β-TRCP to be targeted for protein degradation [12, 25, 26]. However, in our study, although the mutations of both degron motifs on TAZ partially reduce Taxol-induced TAZ degradation (data not shown), the interactions between β-TRCP and Cdk1 phosphorylation mimicking TAZ mutants (TAZ3D and TAZ6D) were not increased compared with TAZ- WT (Figure 6E), strongly suggesting that the major E3 ubiquitin ligase binding to and causing TAZ degradation during Cdk1 phosphorylation of TAZ may not be β-TRCP. Moreover, we have also knocked down β-TRCP or other potential ubiquitin ligases such as FBW7, which has been shown to be involved in antitubulin-induced protein degradation [37]. However, no significant suppression of Taxol-induced TAZ degradation was found after β-TRCP or FBW7 knockdown (data not shown). Therefore, further screening for E3 ubiquitin ligases using a pool of E3 ligase siRNA library may be required to identify the critical E3 ligase(s) responsible for Taxol-induced TAZ degradation.

In this study, we found that within short time of antitubulin drug treatment (<=6 h), only three sites (S90, S105, and T285) of TAZ were phosphorylated by Cdk1, and the rest three sites (T175, T326, and T346) were subsequently phosphorylated with the extension of treatment (6 h-24 h) (Supplementary Figure 1). There are two possibilities that may cause this phenotype. First, Cdk1 activity within 6 hours of drug treatment is not high enough to phosphorylate all the 6 sites on TAZ. And with the extension of treatment time, the activity of Cdk1 may increase to fully phosphorylate all the sites on TAZ. Second possibility is that the first three sites of TAZ may be located on the surface of TAZ and are easy to be targeted. With the phosphorylation of these three sites, TAZ conformation changes, which may expose the rest three sites for Cdk1 phosphorylation and subsequent E3 ligase binding and proteasome degradation. However, in our study, we found that only TAZ6A instead of TAZ3A, can block Cdk1-induced TAZ degradation, highly supporting the first possibility that the phosphorylation of TAZ depending on the activity of Cdk1.

Although in our studies TAZ is found degraded in response to antitubulin drugs through Cdk1 activation, it is also degraded when cells were treated with other DNA damage reagents such as Cisplatin even when all of the Cdk1 phosphorylation sites are mutated (TAZ6A) (Figure 5B). This suggests that TAZ can be degraded through a Cdk1-independent pathway when cells are exposed to other apoptotic stimuli. Since overexpression of TAZ can also cause resistance of tumor cells to other chemotherapeutic drugs such as Doxorubicin [19], it is possible that loss of TAZ may be also critical for cell death induced by other chemotherapeutics. It will be very interesting to identify the kinase(s) phosphorylating and inactivating TAZ in response to other chemotherapeutic drugs.

In conclusion, our studies unveil a novel aspect of TAZ stability regulation by Cdk1 in response to antitubulin drug treatments. The phosphorylation status of TAZ determines its function in antitubulin drug resistance and the Cdk1-TAZ signalling may be potential biomarkers for predicting the sensitivity of cancer patients to antitubulin drugs. Modulating Cdk1 level and its activity to cause TAZ degradation may be a potential target for the treatment of TAZ-induced drug resistance in cancers.

## MATERIALS AND METHODS

### Cell culture, treatments of cells with chemotherapeutic drugs and cycloheximide (CHX)

HEK293T (human kidney cells), HeLa (cervical cancer), and MDA-MB468 and MCF7(human breast cancer) cells were cultured in Dulbecco's Modified Eagle complete medium (DMEM) containing 10% heat-inactivated fetal bovine serum (FBS) (Sigma-Aldrich) and 1% penicillin/streptomycin (P/S); HCT116 (Colon cancer), SK-BR3, and SK-BR3-TR cells were cultured in McCoy's 5A Modified Media (Sigma-Aldrich) with 10% FBS and 1% P/S added. MCF10A (human immortalized mammary epithelial cells) were cultured in DMEM/Nutrient Mixture F12 Ham (Sigma-Aldrich) supplemented with 5% horse serum (HS) (Invitrogen), 200 ug/mL hEGF, 5 mg/mL hydrocortisone, 2 mg/mL insulin, 200 mM L-glutamine, 1mg/mL cholera toxin, and 1% P/S. Cells were maintained at 37°C with 5% CO_2_. Treatments of cells with chemotherapeutic drugs were as described [20].

HeLa cells were cultured to 40% confluency before treating with CHX at concentration of 60 μg/ml alone or together with Taxol (100 nM) for different times (0 h, 4 h, 8 h, 12 h). At each time point, both attached (alive) and floating (dead) cells were lysed with RIPA buffer containing protease inhibitor (PI, Roche) and subjected to western blot analysis.

To test if TAZ phosphorylation and degradation is due to loss of anchorage of cells after antitubulin drug treatment, HeLa cells were plated onto a 60 mm plate coated with Polyhema (20 mg/ml, Sigma) and incubated at 37°C for 24 h with or without adding Taxol (100 nM). Floating cells were collected for protein extraction and western blot analysis. Besides, attached HeLa cells were detached by trypsinization at either 40% or 90% confluency before being lysed for protein extraction and western blot analysis.

### Plasmids construction and transfection

Expression plasmids with various mutations were constructed through site-direct mutagenesis with methods described [38]: TAZ, TAZ mutants and TAZ truncations were cloned into HA-tagged pcDNA3, pGEX4T1 or HA-tagged WPI lentiviral vector. β-TRCP was subcloned into 3 × FLAG-tagged pcDNA3.1-hygro vector. Plasmids were transfected into cells using PolyJet (SignaGen) as described by the manufacture.

### Short interference RNA (siRNA)-mediated gene expression knockdown

These experiments were performed as described [20].

### GST fusion protein production, *in vitro* kinase assay, and phos-tag analysis of TAZ phosphorylation

For GST fusion protein production, TAZ and TAZ6A cloned in pGET4T1 were transformed into BL21 bacteria. Production and purification of GST fusion protein were as described [38]. *In vitro* kinase assay was performed by mixing fusion proteins with or without Cdk1 kinase (Signalchem) in a kinase buffer [1 × PBS (pH 7.4), 20% glycerol, 4 mM MgCl_2_, 10 mM DTT, Protease inhibitor] containing 1 mM of ATP-γ-S (Sigma-Aldrich). Mixed samples were incubated at 30°C for 30 min. Then p-Nitrobenzyl mesylate (PNBM) was added at final concentration of 2.5 mM and the mixed samples were incubated for 1 hour at room temperature. The samples were mixed with equal amount of 2 × SDS loading buffer (0.29M Tris HCl, 8.57% SDS, 30% glycerol, 4.2% Δ-mercaptoethanol, 0.2 mg bromophenol blue) and subjected to western blot analysis. The phosphorylated substrates were detected with anti-thiophosphate ester antibody (Abcam).

For detection of phosphorylated TAZ protein by phos-tag gel, cells transfected with different TAZ constructs and under different culture conditions (e.g., treat with drugs) were washed with TBS buffer [10 mM Tris-HCl (pH 7.5), 100 mM NaCl] before lysed with RIPA [20 mM Tris-HCl (pH 7.5), 150 mM NaCl, 1 mM EDTA, 1 mM EGTA, 1% NP-40, 1% sodium deoxycholate, 2.5 mM sodium pyrophosphate, 1 mM β-glycerophosphate, 1 mM Na_3_VO_4_, 1 μg/ml leupeptin, protease/phosphatase inhibitors]. Protein lysates were mixed with 5× protein loading buffer (0.29M Tris HCl, 8.57% SDS, 30% glycerol, 4.2% Δ-mercaptoethanol, 0.2 mg bromophenol blue) and boiled for 5 min. The sodium dodecyl sulfate polyacrylamide gel electrophoresis (SDS-PAGE) gel with 10 mM MnCl_2_ and 5 mM phos-tag™ Acrylamide ALL-107 (NARD) added in resolving gel were used for running the protein lysates. The gels were soaked in transfer buffer (48 mM Tris, 39 mM glycine, 0.04% SDS, 20% methanol) with 1 mM EDTA added for 10 minutes at room temperature and washed twice with transfer buffer before transfer to polyvinyl difluoride (PVDF) memberane at 4°C. The phosphorylated TAZ and its mutants were detected using anti-HA antibody (1:1000, Abcam).

### Lentiviral production, infection and establishment of stable cell lines

HEK293T cells were cultured in 60 mm tissue culture dishes pre-coated with 0.1 mg/mL poly-L-lysine (PLL) for transfection with packaging plasmid (psPAX), envelop plasmid (pMD2G) and related transfer vectors (e.g. WPI-TAZ-HA, WPI-TAZ3A-HA, WPI-TAZ3D-HA, WPI-TAZ6A-HA, WPI-TAZ6D-HA) using PolyJet reagent according to manufacturer's protocol. Cells were incubated in an incubator with 5% CO_2_ at 37°C overnight. The media were refreshed with DMEM containing 10 mM Sodium Butyrate and incubated for 24 hours. The medium containing lentivirus was directly collected and used to infect MCF10A cells with polybrene at 8 μg/ml.

### RNA extraction and quantitative reverse transcription PCR (qRT-PCR)

MCF10A cells stably expressing TAZ and its mutants (TAZ, TAZ3A, TAZ3D, TAZ6A, TAZ6D) were cultured until 70–80% confluency before RNA extraction through RNA extraction and purification kit from QIAGEN according to the manual. Real time qRT-PCR analysis was performed as described [38].

### Antibodies, western blot and co-immunoprecipitation (co-IP)

Protein extract, western blot and co-IP were performed as described [36]. Antibodies used were as followings: mouse monoclonal anti-TAZ (1:1000) antibody from BD Biosciences; mouse monoclonal anti-β-actin (1:10, 000) and anti-FLAG (M2; 1:500) antibodies from Sigma; rabbit polyclonal anti-HA (1:1000) and anti-Thiophosphate ester (1:1000) antibodies from Abcam; rabbit polyclonal anti-MST1, MST2, LATS1, CyclinB1, Cdk1, cleaved-PARP (c-PARP), BCL-2, and pCdk1 (all diluted at 1:1000) antibodies from Cell Signalling Technology.

### Analysis of cell death by Trypan blue exclusion analysis

Cells were counted and plated at cell density of 40–50% for different drug treatments for 2 or 3 days. After drug treatment, both floating and attached cells were collected either directly or after trypsinization. Then cells were mixed with 4% Trypan blue (Sigma-Aldrich) at ratio of 1:1 and directly subjected to hemocytometer for counting the stained cells and total cell numbers. The ratio of stained cells (dead cells) in total cells was calculated based on the counted cell number. The experiments were repeated at least three times. The mean and standard errors were calculated. The statistical analysis was performed with unpaired student's *t*-test. A *P* value less than 0.05 is considered as significant. “*” indicates *P* value is < 0.05.

## SUPPLEMENTARY FIGURES


