# Supplementary material for: Regulation of sensitivity of tumor cells to antitubulin drugs by Cdk1-TAZ signalling
Source: Oncotarget. 2015 Jun 8;6(26):21906–17. doi: 10.18632/oncotarget.4259 (PMC4673135; doi:10.18632/oncotarget.4259)
Supplement: Supplementary file 1 [file oncotarget-06-21906-s001.pdf]

SUPPLEMENTARY FIGURES

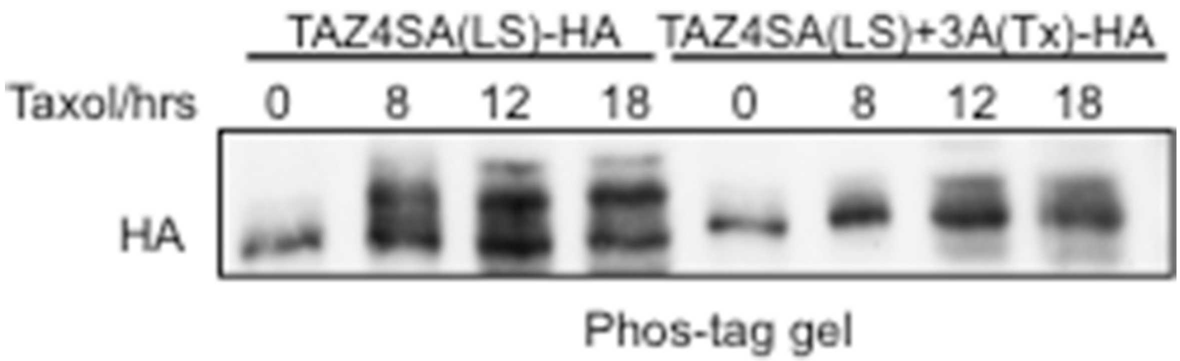

Supplementary Figure 1: Phosphorylations of TAZ by Cdk1 is time dependent. TAZ4SA(LS) and TAZ4SA(LS)+3(Tx) were treated with Texol (100 nM) for 0, 8, 12, 18 hours. Floating cells were collected and lysed for phos-tag detecting phosphorylation of the constructs.

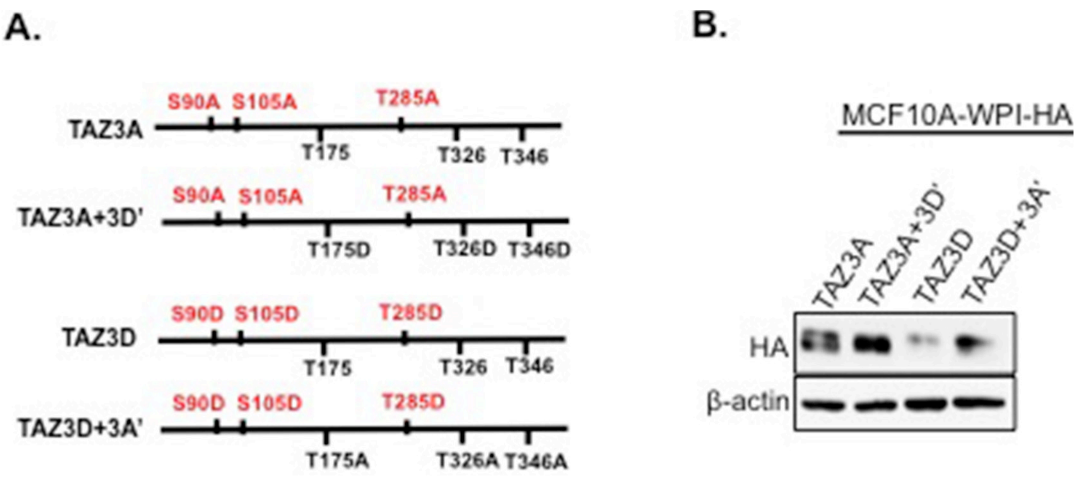

Supplementary Figure 2: The rest three Cdk1 phospohrylation sites on TAZ play a role in TAZ degradation. A. The difference between TAZ3A+3D′, TAZ3D+3A′, TAZ3A and TAZ3D. **B.** Protein expressions of MCF10A stably overexpressing the mutants.
